# Depression is a major risk factor for the development of dementia in people with lower urinary tract symptoms: A nationwide population-based study

**DOI:** 10.1371/journal.pone.0217984

**Published:** 2019-06-07

**Authors:** Ming-Jung Ou, Chun-Che Huang, Yi-Chi Wang, Yung-Liang Chen, Chung-Han Ho, Ming-Ping Wu, Yu-Tung Huang, Chien-Yi Wu, Ping-Jen Chen

**Affiliations:** 1 Department of Family Medicine, Kaohsiung Medical University Hospital, Kaohsiung Medical University, Kaohsiung City, Taiwan; 2 Department of Medical Research, Taichung Veterans General Hospital, Taichung City, Taiwan; 3 Department of Family Medicine, Kaohsiung Municipal Hsiao-Kang Hospital, Kaohsiung Medical University, Kaohsiung City, Taiwan; 4 Department of Family Medicine, Kaohsiung Municipal Ta-Tung Hospital, Kaohsiung Medical University, Kaohsiung City, Taiwan; 5 Department of Medical Research, Chi-Mei Medical Center, Tainan City, Taiwan; 6 Department of Hospital and Health Care Administration, Chia Nan University of Pharmacy and Science, Tainan City, Taiwan; 7 Division of Urogynecology, Department of Obstetrics and Gynecology, Chi Mei Foundation Hospital, Tainan, Taiwan; 8 Department of Obstetrics and Gynecology, College of Medicine, Fu-Jan Catholic University, New Taipei City, Taiwan; 9 Center for Big Data Analytics and Statics, Chang Gung Memorial Hospital, Taoyuan City, Taiwan; 10 School of Medicine, College of Medicine, Kaohsiung Medical University, Kaohsiung, Taiwan; 11 Marie Curie Palliative Care Research Department, Division of Psychiatry, University College London, London, United Kingdom; 12 Palliative Care Center, Chi-Mei Medical Center, Tainan City, Taiwan; Chiba Daigaku, JAPAN

## Abstract

**Background/Objectives:**

Studies have shown a strong relationship between depression and dementia. Lower urinary tract symptoms (LUTS) were reported to be independently associated with depression and dementia. However, the relationship between depression and cognitive dysfunction in patients with LUTS is not well characterized.

**Method:**

We conducted a matched cohort study by using a one-million population-based dataset in Taiwan. A total of 15,944 patients with LUTS aged 50 or older were included from 2001 to 2005 and followed up until their death or the end of 2012. During the follow-up period, 1958 cases developed depression subsequently and were defined as the study group. 7832 patients without depression were then identified as control group, matching by age, gender, insurance premium, status of catastrophic illness certificate, and the index year in a 1:4 ratio. The primary outcome was the onset of dementia. LUTS, depression, dementia, and other comorbidities were defined by the International Classification of Disease, 9th Revision, Clinical Modification coding system. Cox hazards models and Aalen Johansen curves were applied to measure the influence of depression on the risk of dementia in patients with LUTS.

**Results:**

The crude incidence of depression among people with LUTS was 12.3%. The incidence of dementia in the depression group was significantly higher than that in the control group (12.2% versus 8.9%; P < 0.001). Depression was associated with a significantly greater risk of subsequent dementia after adjusted for socioeconomic status, number of outpatient visits and multiple comorbidities (adjusted hazard ratio: 1.32; 95% confidence interval: 1.13–1.54).

**Conclusions:**

Depression is a major risk factor for the onset of subsequent dementia in patients with LUTS. Early screening and interventions for depression in patients with LUTS may be important to maintain cognitive function.

## Introduction

Chronic lower urinary tract symptoms (LUTS) are common in the older population and are associated with an increased risk of functional decline, falls, or diseases, such as metabolic syndrome and cardiovascular events [1–4]. LUTS also have a negative impact on health-related quality of life (HRQoL) [5, 6], and increase care-giver burden [7], dependency, and healthcare costs [8]. The adverse effects of LUTS on HRQoL were shown to be more than those of common comorbid illnesses, including diabetes, hypertension, and gout [9, 10]. According to a study, the annual total cost of LUTS in the United States was 66 billion dollars in 2007, which is projected to increase to 82.6 billion dollars in 2020 [11]. A cross-sectional study in Taiwan showed an increasing prevalence of LUTS patients seeking healthcare from 2.31% in 2000 to 3.84% in 2009 [12]. Another cross-sectional study in Taiwan based on an internet questionnaire in 2015 showed higher prevalence in both men and women aged above forty years old. By using International Continence Society criteria, LUTS was self-reported by 60% of men and 57% of women [13]. Therefore, identifying and managing risk factors for major comorbidities and the impaired HRQoL of this large population of patients with LUTS is a pressing issue.

Depression adversely affects HRQoL; moreover, it was shown to be associated with LUTS [14–16]. In a population-based study in Korea, 11.5% of individuals with LUTS were found to be affected by depression (as assessed by CES-D scale) as compared to 2.9% of those without LUTS [17]. A Chinese longitudinal study showed that men with moderate to severe LUTS were 2.5 times more likely to have depressive symptoms than men with mild or no LUTS [18].

Our previous longitudinal study showed that LUTS may increase the risk of dementia. The adjusted hazard ratio(HR) for dementia was 1.61 in patients with LUTS as compared to those without LUTS [19]. Several studies have shown a strong link between depression and dementia [20–23]. Patients with depression showed a two-fold higher risk of incident dementia [24], and a single depressive episode was associated with a 14% increase in risk for all-cause dementia [25]. However, the association between depression and cognitive dysfunction in individuals with LUTS is not well characterized.

In this cohort study on nationwide population-based health insurance claims data in Taiwan, we assessed the hypothesis that depression may be associated with a higher risk of subsequent dementia in subjects with LUTS.

## Materials and methods

### Data source

This population-based cohort study used data from the Longitudinal Health Insurance Database (LHID), which had one million beneficiaries who were randomly selected from the National Health Insurance Research Database (NHIRD), Taiwan. More than 99% of Taiwanese citizens are covered by the National Health Insurance (NHI). All claims-related data for healthcare services are collected and encrypted in the NHIRD. The distribution of age, sex, and average insured payroll-related premiums did not differ between the LHID sample and all NHIRD enrollees. The LHID includes information on outpatient visits, hospital admissions, prescriptions, disease status, and demographic data. To protect patient confidentiality, all their identification numbers and medical institutions were encrypted and maintained by the National Health Research Institutes of Taiwan prior to data release. The diagnostic and procedural codes in the LHID are listed according to the International Classification of Disease, 9th Revision, Clinical Modification (ICD-9-CM) coding system. The data underlying this study is from the NHIRD, which has been transferred to the Health and Welfare Data Science Center (HWDC). Interested researchers can obtain the data through formal application to the HWDC, Department of Statistics, Ministry of Health and Welfare, Taiwan. The Institutional Review Board of the Chi Mei Medical Center approved the study protocol (IRB No. 10708-E01). The requirement for written informed consent was waived owing to the retrospective study design.

### Definition of LUTS

We identified patients who used outpatient services or who made hospitalization claims during the study period with the following ICD-9-CM code categories: (a) storage symptoms, including hypertonicity of the bladder (ICD-9-CM code 596.51), stress urinary incontinence in women (625.6) and men (788.32), urgent incontinence (788.31), frequency and polyuria (788.4), nocturnal enuresis (788.36), nocturia (788.43), and mixed incontinence (788.33); (b) voiding symptoms, including retention of urine (788.2), splitting and slowing of urine stream (788.6), and post-void dribbling (788.35); and (c) benign prostate hyperplasia (BPH; 600.0–600.9).

### Definition of depression

Depression was diagnosed as per the following categories: major depressive disorder (296.20–296.25); major depressive disorder, recurrent episode (296.30–296.35); dysthymic disorder (300.4); and depressive disorder not elsewhere classified (311) [26]. The following codes for antidepressant drugs were identified from the LHID based on the Anatomical Therapeutic Chemical system of medications: N05AN01, N06AA02, N06AA04, N06AA09, N06AA12, N06AA14, N06AA21, N06AB03, N06AB04, N06AB05, N06AB06, N06AB08, N06AB10, N06AG02, N06AX03, N06AX05, N06AX09, N06AX11, N06AX12, N06AX16, N06AX17, N06AX21, N06AX22, N06CA01, and N06CA02 [27].

According to the 2016 guidelines of the Canadian Network for Mood and Anxiety Treatment [28], non-pharmacological treatment is the first-line therapy for mild depression. Pharmacological treatment should be considered in some situations, including patients with a major depressive episode of moderate or greater severity, patient preference, previous response to antidepressants, or non-response to non-pharmacological interventions [28]. Based on this guideline, patients who had received antidepressant drugs twice may have moderate to severe depression. Therefore, we identified patients with confirmed depression as those who (1) had a diagnosis of depression and (2) received at least two antidepressant prescriptions within one year preceding the index date. The date of the first depression code was considered as the index date of entry. However, <2 antidepressant prescriptions within a one-year span were defined as non-antidepressant use.

### Identification of patients

We recruited patients with LUTS who met either of the following criteria during 2001–2005: (1) at least three outpatient service claims with LUTS codes within one year after the first LUTS code; or (2) any hospitalization due to LUTS.

**Fig 1:**
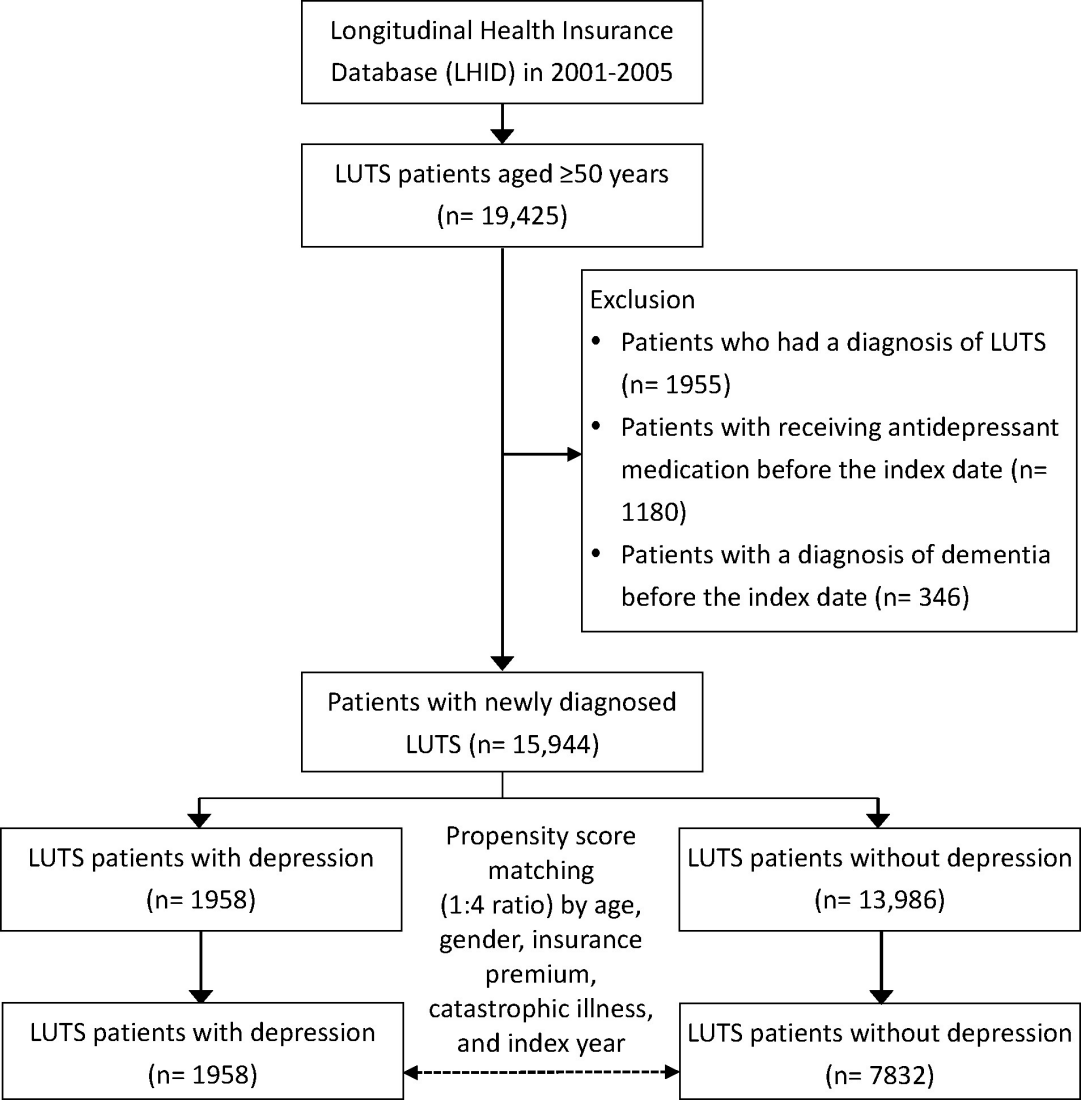
Schematic illustration of the study population and patient-selection criteria.

The exclusion criteria were (1) any one-time ICD-9-CM code of LUTS or dementia (290.0–290.4, 331.0) prior to inclusion and (2) any antidepressant prescription before the first diagnosis of LUTS. Previous studies had indicated that age, gender, and socioeconomics were the important factors of dementia [29–31]. To reduce potential selection bias and to balance patients with and without medication, we randomly selected a control cohort to match each case from the eligible source population using propensity score matching based on age, gender, insurance premium, status of catastrophic illness certificate, and the index year at a ratio of 1:4. The cost of insurance premium for individuals are calculated based on the monthly income they report to the Taiwan’s National Health Insurance Administration (NHIA) under NHI scheme. Patients who have severe disease in various categories including autoimmune disease such as systemic lupus erythematosus or rheumatoid arthritis, cancers, chronic psychiatric diseases such as schizophrenic disorders, affective psychosis, etc., haemodialysis, or congenital disorders such as congenital muscular dystrophy, congenital hypothyroidism and type I diabetes mellitus, etc., are eligible for catastrophic illness certificates after the review and approval by the NHIA. Eligible patients with a catastrophic illness certificate are entitled to a waiver for medical co-payments, including for outpatient visits and inpatient admissions. The status of catastrophic illness certificate was chosen as the surrogate marker of comorbidity severity for matching and the index year was also matched for avoiding the immortal time bias. A schematic illustration of the study population and patient-selection criteria is shown in Fig 1.

### Covariates

The following covariates were selected: age at LUTS diagnosis, sex, status of catastrophic illness certificate, socioeconomic factors by using insurance premium, numbers of outpatient visits, and comorbidities. Age was categorized into four groups: 50−59, 60−69, 70−79, and ≥80 years. The cost of insurance premium for each patient was determined by his/her work salary per month in Taiwan dollars (TWD), and premiums were classified into five groups: ≥ 45,801, 28,801–45,800, 15,841–28,800, <15,840, and dependents. The dependent group referred to those family members who were not employed or paid with a salary, such as housewives, elderly people, and students. Numbers of outpatient visits were defined as average of annual outpatient visits per person after index time. The comorbidity status of patients with LUTS was determined by ICD-9-CM codes: diabetes mellitus (DM; 250), hypertension (HTN; 401‒405), hyperlipidemia (272), coronary artery disease (CAD; 410−414), cerebrovascular disease (430−438), and atrial fibrillation (427.31).

### Follow-up and outcome measures

The primary outcome of this study was the first diagnosis of dementia: senile and presenile dementia (ICD-9-CM codes 290.0‒290.3), vascular dementia (290.4), and Alzheimer’s disease (331.0). The diagnosis of dementia had to fit either of the following criteria: (1) at least three outpatient service claims with the codes of dementia within one year after the first dementia code; or (2) any single hospitalization with dementia among the 5 principal claims diagnosis codes. The identified patients were followed up until death or the end of 2012.

### Statistical analysis

The distribution of demographic characteristics, catastrophic illness certificates, insurance premium, numbers of outpatient visits and comorbid conditions before and after matching between the two cohorts was examined using chi-squared or Fisher’s exact tests for categorical variables and Student’s *t* test for continuous variables. A propensity score matching (PSM) approach was applied to minimize the potential selection bias between the cohorts with LUTS with and without depression.

In addition, the cumulative incidence of dementia for cohorts with LUTS with and without depression was estimated by using Aalen-Johansen estimator. Fine-Gray models were applied for between group differences. Univariate and multivariable Cox proportional hazard models with the robust sandwich variance estimator were used to evaluate the depression status of patients with LUTS associated with the development of dementia. Hazard ratios (HRs) with 95% confidence intervals (CIs) were calculated. P values <0.05 were considered statistically significant. All statistical analyses were performed using SAS version 9.4 (SAS Institute, Cary, NC, USA).

## Results

A total of 15,944 patients with newly diagnosed LUTS were included in our cohort study and 1,958 patients occurred depression afterward (study group). The crude incidence of depression among people with LUTS was 12.3%. In the next step, 7,832 patients without depression were identified as control group after matching with confounding factors (Fig 1).

The depression group had a significantly lower prevalence of HTN and DM, but had higher number of outpatient visits (Table 1). A significantly (P < 0.001) greater proportion of patients in the depression group (12.3%) developed dementia during follow-up as compared to that in the control group (8.9%). The follow-up time from the diagnosis of LUTS to the onset of dementia is 9.0±2.2 years (mean±standard deviation, SD) in the matched cohort.

**Table 1:** Basic characteristics of patients with lower urinary tract symptoms before and after propensity score matching.

Characteristics	Before matching					After matching				
	LUTS with depression (n = 1958)		LUTS without depression (n = 13,986)		P	LUTS with depression (n = 1958)		LUTS without depression (n = 7832)		P
	n	(%)	n	(%)		n	(%)	n	(%)	
Age (years), mean	65.0	(10.2)	65.74	(10.7)	0.003	65.0	(10.2)	65.1	(10.2)	0.717
50~60	569	(29.1)	3544	(25.3)	<0.001	569	(29.1)	2278	(29.1)	0.997
60~70	623	(31.8)	4477	(32.0)		623	(31.8)	2506	(32.0)	
70~80	590	(30.1)	4247	(30.4)		590	(30.1)	2355	(30.1)	
>80	176	(9.0)	1718	(12.3)		176	(9.0)	693	(8.8)	
Gender					<0.001					0.907
Male	1466	(74.9)	11,729	(83.9)		1466	(74.9)	5875	(75.0)	
Female	492	(25.1)	2257	(12.3)		492	(25.1)	1957	(25.0)	
Insurance premium (TWD)					0.413					0.954
≥45,801	113	(5.7)	911	(6.5)		113	(5.7)	443	(5.7)	
28,801–45,800	215	(11.0)	1510	(10.8)		215	(11.0)	881	(11.2)	
15,841–28,800	775	(39.6)	5679	(40.6)		775	(39.6)	3098	(39.6)	
<15,840	448	(22.9)	2980	(21.3)		448	(22.9)	1739	(22.2)	
Dependent	407	(20.8)	2906	(20.8)		407	(20.8)	1671	(21.3)	
Number of outpatient visits per year, mean	30.0	(20.6)	26.0	(19.2)	<0.001	30.0	(20.6)	26.7	(19.5)	<0.001
Catastrophic illness certificate	299	(15.3)	2035	(14.6)	0.394	299	(15.3)	1111	(14.2)	0.221
Hypertension	182	(9.3)	1738	(12.4)	<0.001	182	(9.3)	931	(11.9)	0.001
Diabetes	100	(5.1)	912	(6.5)	0.015	100	(5.1)	509	(6.5)	0.021
Coronary artery disease	32	(1.6)	337	(2.4)	0.03	32	(1.6)	178	(2.3)	0.082
Hyperlipidemia	27	(1.4)	204	(1.5)	0.841	27	(1.4)	106	(1.3)	0.913
Cerebrovascular disease	42	(2.2)	355	(2.5)	0.315	42	(2.2)	188	(2.4)	0.559
Atrial fibrillation	2	(0.1)	39	(0.3)	0.228	2	(0.1)	20	(0.3)	0.287
Dementia	239	(12.2)	1267	(9.1)	<0.001	239	(12.2)	697	(8.9)	<0.001

LUTS, lower urinary tract symptoms; SD, standard deviation; TWD, Taiwan dollar

**Fig 2:**
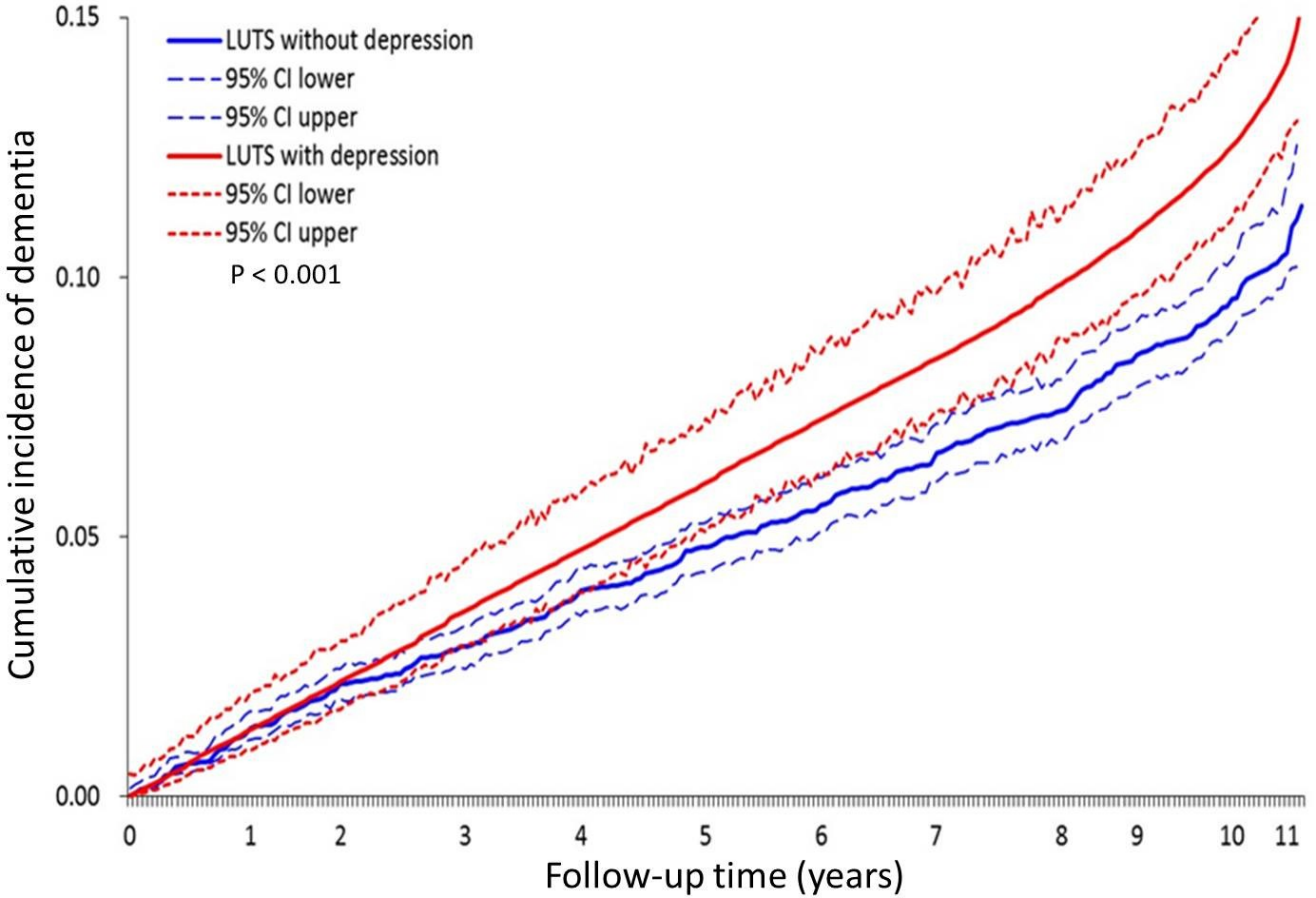
Difference of the cumulative incidence of dementia between patients with lower urinary tract symptoms (LUTS) with and without depression and 95% confidence band. P< 0.001.

In the Cox regression model, age and cerebrovascular disease were still the most critical factors associated with the onset of dementia (Table 2). Depression was associated with a significantly greater risk of subsequent dementia (adjusted HR: 1.32; 95% CI: 1.13–1.54), which is similar to the risk contributed by diabetes or catastrophic illness certificate after adjusting for other confounders (Table 2). Low socioeconomic status and higher frequency of outpatient clinics visits were associated with higher risk of dementia, whereas CAD showed the opposite relationship. A subgroup analysis of demographic data and the Cox regression model between patients with and without BPH was also performed and is presented in the supporting information (S1–S4 Tables). The cumulative incidence of dementia in patients with LUTS with depression was significantly higher than that in patients with LUTS without depression (Fig 2).

**Table 2:** Cox proportional hazard regression analyses for the risk of dementia among patients with lower urinary tract symptoms.

	Univariate model			Multivariable model		
	HR	(95% CI)	P	HR	(95% CI)	P
Depression	1.33	(1.14‒1.56)	<0.001	1.32	(1.13‒1.54)	<0.001
Age (years)						
50~60	0.33	(0.25‒0.44)	<0.001	0.36	(0.27‒0.49)	<0.001
60~70	1.00			1.00		
70~80	2.12	(1.79‒2.51)	<0.001	1.87	(1.57‒2.23)	<0.001
>80	2.74	(2.22‒3.39)	<0.001	2.61	(2.12‒3.22)	<0.001
Gender						
Male	1.00			1.00		
Female	0.92	(0.78‒1.08)	0.296	1.09	(0.92‒1.30)	0.287
Insurance premium (TWD)						
≥45,801	0.31	(0.19‒0.50)	<0.001	0.53	(0.33‒0.86)	0.011
28,801–45,800	0.51	(0.38‒0.70)	<0.001	0.89	(0.65‒1.21)	0.418
15,841–28,800	1.00			1.00		
<15,840	1.78	(1.53‒2.07)	<0.001	1.22	(1.03‒1.43)	0.019
Dependent	1.22	(1.02‒1.45)	0.033	1.17	(0.97‒1.40)	0.102
Number of outpatient visits	1.01	(1.01‒1.02)	<0.001	1.01	(1.00‒1.01)	<0.001
Catastrophic illness certificate	1.47	(1.25‒1.72)	<0.001	1.25	(1.07‒1.48)	0.006
Hypertension	1.39	(1.17‒1.66)	<0.001	1.13	(0.94‒1.36)	0.192
Diabetes	1.51	(1.21‒1.88)	<0.001	1.34	(1.05‒1.70)	0.019
Coronary artery disease	0.89	(0.55‒1.43)	0.635	0.58	(0.36‒0.95)	0.029
Hyperlipidemia	1.14	(0.68‒1.90)	0.625	1.21	(0.73‒1.99)	0.469
Cerebrovascular disease	2.42	(1.72‒3.41)	<0.001	1.92	(1.35‒2.73)	<0.001
Atrial fibrillation	2.58	(1.10‒6.02)	0.029	1.98	(0.84‒4.63)	0.117

CI, confidence interval; HR, hazard ratio; LUTS, TWD, Taiwan dollar.

## Discussion

In this nationwide population-based cohort study, depression was identified as a major risk factor for the subsequent development of dementia in patients with LUTS after adjusting for socioeconomic status, number of outpatient visits, and multiple comorbidities. To our knowledge, this is the first study in Asia that explored the link between depression and the occurrence of dementia in patients with LUTS. Dementia imposes a substantial burden on patients and the society [32]; therefore, identifying its risk factors is imperative [33]. Our findings suggest that the early detection of and intervention for depression may help prevent the development of dementia in patients with LUTS.

Chronic diseases, such as DM, CAD, stroke, and chronic obstructive pulmonary disease, were shown to be associated with depression [34, 35]. Previous cross-sectional [1, 14, 15, 36] and longitudinal studies [18] have shown a significant association between LUTS and depression. Men with storage LUTS were shown to be 2.77 times more likely to develop depression [16]. In a longitudinal study in Korea, patients with severe LUTS were shown to be at a 3.9 times higher risk of depression than those with none to mild LUTS [37].

There are several mechanisms by which LUTS can lead to depression. First, LUTS can cause embarrassment, discourage daily activity, and hinder social participation [1]. Men with LUTS show decreased sexual activity, which can also lead to depression [17]. Second, LUTS, especially nocturia, may lead to depression through sleep disturbances and circadian misalignment [38]. Third, systemic inflammation is a known risk factor for both LUTS [39] and depression [40], and may partially mediates the association between depression and LUTS [41]. Elevated levels of proinflammatory cytokines often precede a depressive episode and may persist even after the remission of depression [42], which may be associated with subsequent dementia. Fourth, neurotransmitters are involved in the physiological process of LUTS and depression. Recent experiments suggest that serotonin has central nervous system effects that impair lower urinary tract function [43]. The correlation between LUTS and depression may indeed be bi-directional, since depression may affect LUTS by enhancing the activation of the sympathetic nervous system [44].

Depression and dementia are common neuropsychiatric disorders in later life. They share common etiological factors, such as inflammation, vascular changes, and vascular risk factors [45]. However, the exact mechanism of the interconnection is still obscure. Depression may be both a prodrome and a risk factor for dementia [33]. A literature review by Bennett et al. mentioned that early life depression is associated with an increased risk of dementia, while late life depression is a prodrome of dementia [43].

Studies have suggested that long-term antidepressant therapy in older patients with depression may reduce the risk of some types of dementia [46]. Some antidepressants may have a neuroprotective effect [46], which improve memory and cognitive function, but some may have the opposite effect [47]. A retrospective case–control study in Taiwan demonstrated that the risk of dementia in patients with major depression decreased with tricyclic antidepressants, but increased with other antidepressants [48]. Evaluation of the utilization pattern and individual effect of antidepressant therapy on the risk of dementia in patients with LUTS needs to be explored in a future study.

Our previous study showed that LUTS increase the risk of dementia [19]. In this study, age and cerebrovascular disease remained the most critical factor for dementia. The high HR of cerebrovascular disease is in line with current evidence but might be partially attributed to physicians, such as neurologists or geriatricians, who specialized in the relationship between cerebrovascular disease and dementia, and noted to the onset of dementia. The patient group with highest insurance premium showed significantly decreased risk for further development of dementia, whereas the group with lowest insurance premium had an increased risk of dementia, and the dependent group also had a trend toward higher risk. Studies have suggested that low socioeconomic status may contribute to increased dementia incidence [49]. This might be explained because people who have higher economic status usually have more access to better educational status and a more mentally stimulating environment, which may contribute to higher cognition reserve.

### Strength and limitation

The strengths of this study include the use of a large group of nationwide population-based samples and its longitudinal cohort study design. Depression was diagnosed based on both the ICD-9-CM codes and antidepressant prescriptions, which are more reliable than diagnosis based on questionnaires used in other studies. The factors of socioeconomic status and medical utilization had been considered and subgroup analysis was also performed. However, our study had some limitations. First, we did not evaluate the duration and cumulative dose of antidepressants, which may affect further dementia prevalence. Second, some demographic data and lifestyle factors, such as smoking, alcohol use, exercise, and social interaction could not be obtained from the NHIRD. These are potential risk factors for dementia and depression. Third, we cannot classify the severity of LUTS, depression, and dementia due to the nature of the dataset, which may have different pathophysiological basis. Fourth, the diagnoses of LUTS, depression and dementia have not been comprehensively validated in the NHIRD. Several validation studies of various disease in NHIRD such as stroke and myocardial infarction, however, generally reported high positive predictive value [50, 51]. To enhance the accuracy of diagnosis, we used relatively strict inclusion criteria requiring at least three outpatient visits or one admission record, and this might underestimate the incidence of diseases.

## Conclusions

Depression was associated with an increased risk of dementia in patients with LUTS. LUTS, depression, and dementia increase the burden on patients and care-givers. Early screening for depression among patients with LUTS and timely interventions, such as psychotherapy, behavioral therapy, and pharmacological therapy, may help reduce or even prevent cognitive impairment in these patients. Future studies should evaluate the association of clinical severity, subtypes of depression and dementia, and the effect of antidepressant use to understand the mechanisms that may have contributed to the results of our study.

## Supporting information

S1 Table Basic characteristics of patients with lower urinary tract symptoms excluding benign prostatic hyperplasia before and after propensity score matching. (DOCX)

S2 Table Cox proportional hazard regression analyses for the risk of dementia among patients with lower urinary tract symptoms excluding benign prostatic hyperplasia. (DOCX)

S3 Table Basic characteristics of patients with benign prostatic hyperplasia before and after propensity score matching. (DOCX)

S4 Table Cox proportional hazard regression analyses for the risk of dementia among patients with benign prostatic hyperplasia. (DOCX)
