# Supplementary material for: Depression is a major risk factor for the development of dementia in people with lower urinary tract symptoms: A nationwide population-based study
Source: PLoS One. 2019 Jun 7;14(6):e0217984. doi: 10.1371/journal.pone.0217984 (PMC6555508; doi:10.1371/journal.pone.0217984)
Supplement: S1 Table — (DOCX) [file pone.0217984.s001.docx]

**S1 Table. Basic characteristics of patients with lower urinary tract symptoms excluding benign prostatic hyperplasia before and after propensity score matching**

| Characteristics | Before matching | | | | |  | After matching | | | | |
| --- | --- | --- | --- | --- | --- | --- | --- | --- | --- | --- | --- |
|  | LUTS with depression  (n = 947) | | LUTS without depression  (n = 6534) | | P |  | LUTS with depression  (n = 947) | | LUTS without depression  (n = 3788) | | P |
|  | n | (%) | n | (%) |  |  | n | (%) | n | (%) |  |
| Age (years), mean (SD) | 63.7 | (11.0) | 65.0 | (12.2) | 0.005 |  | 63.7 | (11.0) | 64.1 | (11.1) | 0.371 |
| 50~60 | 295 | (31.2) | 1682 | (25.8) | <0.001 |  | 295 | (31.2) | 1171 | (30.9) | 0.999 |
| 60~70 | 291 | (30.7) | 1889 | (28.9) |  |  | 291 | (30.7) | 1173 | (31.0) |  |
| 70~80 | 253 | (26.7) | 1837 | (28.1) |  |  | 253 | (26.7) | 1012 | (26.7) |  |
| >80 | 108 | (11.4) | 1126 | (17.2) |  |  | 108 | (11.4) | 432 | (11.4) |  |
| Gender |  |  |  |  | <0.001 |  |  |  |  |  | 1.000 |
| Male | 456 | (48.2) | 4277 | (65.5) |  |  | 456 | (48.2) | 1824 | (48.2) |  |
| Female | 491 | (58.8) | 2257 | (34.5) |  |  | 491 | (58.8) | 1964 | (58.8) |  |
| Insurance premium (TWD) |  |  |  |  | 0.987 |  |  |  |  |  | 0.223 |
| ≥45,801 | 39 | (4.1) | 286 | (4.4) |  |  | 39 | (4.1) | 151 | (4.0) |  |
| 28,801–45,800 | 83 | (8.8) | 600 | (9.2) |  |  | 83 | (8.8) | 342 | (9.0) |  |
| 15,841–28,800 | 436 | (46.0) | 2973 | (45.5) |  |  | 436 | (46.0) | 1793 | (47.3) |  |
| <15,840 | 173 | (18.3) | 1188 | (18.2) |  |  | 173 | (18.3) | 576 | (15.2) |  |
| Dependent | 216 | (22.8) | 1487 | (22.7) |  |  | 216 | (22.8) | 926 | (24.5) |  |
| Number of outpatient visits per year, mean (SD) | 32.7 | (20.6) | 27.9 | (19.9) | <0.001 |  | 32.7 | (20.6) | 29.0 | (19.7) | <0.001 |
| Catastrophic illness certificate | 162 | (17.1) | 1081 | (16.5) | 0.674 |  | 162 | (17.1) | 600 | (15.8) | 0.348 |
| Hypertension | 113 | (11.9) | 1125 | (17.2) | <0.001 |  | 113 | (11.9) | 529 | (14.0) | 0.111 |
| Diabetes | 61 | (6.4) | 651 | (10.0) | <0.001 |  | 61 | (6.4) | 356 | (9.4) | 0.004 |
| Coronary artery disease | 27 | (2.9) | 277 | (4.2) | 0.043 |  | 27 | (2.9) | 129 | (3.4) | 0.476 |
| Hyperlipidemia | 15 | (1.6) | 142 | (2.2) | 0.275 |  | 15 | (1.6) | 68 | (1.8) | 0.782 |
| Cerebrovascular disease | 35 | (3.7) | 308 | (4.7) | 0.183 |  | 35 | (3.7) | 150 | (4.0) | 0.779 |
| Atrial fibrillation | 2 | (0.2) | 37 | (0.6) | 0.224 |  | 2 | (0.2) | 15 | (0.4) | 0.551 |
| Dementia | 119 | (12.6) | 575 | (8.8) | <0.001 |  | 119 | (12.6) | 337 | (8.9) | <0.001 |

LUTS, lower urinary tract symptoms; SD, standard deviation; TWD, Taiwan dollar
