# Supplementary material for: Depression is a major risk factor for the development of dementia in people with lower urinary tract symptoms: A nationwide population-based study
Source: PLoS One. 2019 Jun 7;14(6):e0217984. doi: 10.1371/journal.pone.0217984 (PMC6555508; doi:10.1371/journal.pone.0217984)
Supplement: S2 Table — (DOCX) [file pone.0217984.s002.docx]

**S2 Table. Cox proportional hazard regression analyses for the risk of dementia among patients with lower urinary tract symptoms excluding benign prostatic hyperplasia**

|  | Univariate model | | |  | Multivariable model | | |
| --- | --- | --- | --- | --- | --- | --- | --- |
|  | HR | (95% CI) | P |  | HR | (95% CI) | P |
| Depression | 1.38 | (1.11‒1.73) | 0.004 |  | 1.39 | (1.12‒1.73) | 0.003 |
| Age (years) |  |  |  |  |  |  |  |
| 50~60 | 0.33 | (0.23‒0.47) | <0.001 |  | 0.38 | (0.26‒0.55) | <0.001 |
| 60~70 | 1.00 |  |  |  | 1.00 |  |  |
| 70~80 | 2.10 | (1.66‒2.66) | <0.001 |  | 1.92 | (1.51‒2.45) | <0.001 |
| >80 | 1.98 | (1.46‒2.69) | <0.001 |  | 2.11 | (1.55‒2.87) | <0.001 |
| Gender |  |  |  |  |  |  |  |
| Male | 1.00 |  |  |  | 1.00 |  |  |
| Female | 0.87 | (0.73‒1.05) | 0.141 |  | 1.13 | (0.90‒1.41) | 0.299 |
| Insurance premium (TWD) |  |  |  |  |  |  |  |
| ≥45,801 | 0.17 | (0.05‒0.54) | 0.003 |  | 0.26 | (0.08‒0.85) | 0.026 |
| 28,801–45,800 | 0.38 | (0.22‒0.65) | <0.001 |  | 0.65 | (0.38‒1.11) | 0.117 |
| 15,841–28,800 | 1.00 |  |  |  | 1.00 |  |  |
| <15,840 | 1.55 | (1.22‒1.98) | <0.001 |  | 1.13 | (0.87‒1.46) | 0.366 |
| Dependent | 1.35 | (1.08‒1.70) | 0.009 |  | 1.31 | (1.04‒1.64) | 0.021 |
| Number of outpatient visits | 1.01 | (1.01‒1.02) | <0.001 |  | 1.01 | (1.00‒1.01) | <0.001 |
| Catastrophic illness certificate | 1.35 | (1.07‒1.71) | 0.010 |  | 1.20 | (0.94‒1.52) | 0.145 |
| Hypertension | 1.51 | (1.19‒1.91) | <0.001 |  | 1.19 | (0.90‒1.57) | 0.218 |
| Diabetes | 1.58 | (1.19‒2.10) | 0.002 |  | 1.31 | (0.98‒1.77) | 0.073 |
| Coronary artery disease | 0.99 | (0.62‒1.60) | 0.980 |  | 0.68 | (0.42‒1.09) | 0.109 |
| Hyperlipidemia | 1.31 | (0.72‒2.39) | 0.380 |  | 1.37 | (0.75‒2.52) | 0.306 |
| Cerebrovascular disease | 2.21 | (1.52‒3.21) | <0.001 |  | 1.70 | (1.15‒2.52) | 0.008 |
| Atrial fibrillation | 2.72 | (1.05‒7.05) | 0.040 |  | 2.40 | (0.92‒6.24) | 0.073 |

CI, confidence interval; HR, hazard ratio; TWD, Taiwan dollar
