# Supplementary material for: Depression is a major risk factor for the development of dementia in people with lower urinary tract symptoms: A nationwide population-based study
Source: PLoS One. 2019 Jun 7;14(6):e0217984. doi: 10.1371/journal.pone.0217984 (PMC6555508; doi:10.1371/journal.pone.0217984)
Supplement: S3 Table — (DOCX) [file pone.0217984.s003.docx]

**S3 Table. Basic characteristics of patients with benign prostatic hyperplasia before and after propensity score matching**

| Characteristics | Before matching | | | | |  | After matching | | | | |
| --- | --- | --- | --- | --- | --- | --- | --- | --- | --- | --- | --- |
|  | BPH with depression  (n = 1011) | | BPH without depression  (n = 7452) | | P |  | BPH with depression  (n = 1011) | | BPH without depression  (n = 4044) | | P |
|  | n | (%) | n | (%) |  |  | n | (%) | n | (%) |  |
| Age (years), mean (SD) | 66.2 | (9.2) | 66.6 | (9.2) | 0.279 |  | 66.2 | (9.2) | 66.7 | (9.2) | 0.191 |
| 50~60 | 274 | (27.1) | 1862 | (25.0) | 0.215 |  | 274 | (27.1) | 1000 | (24.7) | 0.298 |
| 60~70 | 332 | (32.9) | 2588 | (34.7) |  |  | 332 | (32.9) | 1371 | (33.9) |  |
| 70~80 | 337 | (33.3) | 2410 | (32.3) |  |  | 337 | (33.3) | 1351 | (33.4) |  |
| >80 | 68 | (6.7) | 592 | (8.0) |  |  | 68 | (6.7) | 322 | (8.0) |  |
| Gender |  |  |  |  | NA |  |  |  |  |  | NA |
| Male | 1011 | (100.0) | 7452 | (100.0) |  |  | 1011 | (100.0) | 4044 | (100.0) |  |
| Female | – | – | – | – |  |  | – | – | – | – |  |
| Insurance premium (TWD) |  |  |  |  | 0.121 |  |  |  |  |  | 0.232 |
| ≥45,801 | 74 | (7.3) | 625 | (8.4) |  |  | 74 | (7.3) | 316 | (7.8) |  |
| 28,801–45,800 | 132 | (13.1) | 910 | (12.2) |  |  | 132 | (13.1) | 504 | (12.5) |  |
| 15,841–28,800 | 339 | (33.5) | 2706 | (36.3) |  |  | 339 | (33.5) | 1470 | (36.3) |  |
| <15,840 | 275 | (27.2) | 1792 | (24.0) |  |  | 275 | (27.2) | 973 | (24.1) |  |
| Dependent | 191 | (18.9) | 1419 | (19.1) |  |  | 191 | (18.9) | 781 | (19.3) |  |
| Number of outpatient visits per year, mean (SD) | 27.5 | (20.3) | 24.3 | (18.3) | <0.001 |  | 27.5 | (20.3) | 24.4 | (18.6) | <0.001 |
| Catastrophic illness certificate | 137 | (13.6) | 954 | (12.8) | 0.515 |  | 137 | (13.6) | 515 | (12.7) | 0.495 |
| Hypertension | 69 | (6.8) | 613 | (8.2) | 0.139 |  | 69 | (6.8) | 345 | (8.5) | 0.083 |
| Diabetes | 39 | (3.9) | 261 | (3.5) | 0.586 |  | 39 | (3.9) | 141 | (3.5) | 0.569 |
| Coronary artery disease | 5 | (0.5) | 60 | (0.8) | 0.439 |  | 5 | (0.5) | 32 | (0.8) | 0.412 |
| Hyperlipidemia | 12 | (1.2) | 62 | (0.8) | 0.277 |  | 12 | (1.2) | 31 | (0.8) | 0.184 |
| Cerebrovascular disease | 7 | (0.7) | 47 | (0.6) | 0.832 |  | 7 | (0.7) | 24 | (0.6) | 0.657 |
| Atrial fibrillation | 0 | (0.0) | 2 | (0.03) | 1.000 |  | 0 | (0.0) | 2 | (0.05) | 1.000 |
| Dementia | 120 | (11.9) | 692 | (9.3) | 0.010 |  | 120 | (11.9) | 380 | (9.4) | 0.021 |

BPH, benign prostatic hyperplasia; SD, standard deviation; TWD, Taiwan dollar
