# Supplementary material for: Depression is a major risk factor for the development of dementia in people with lower urinary tract symptoms: A nationwide population-based study
Source: PLoS One. 2019 Jun 7;14(6):e0217984. doi: 10.1371/journal.pone.0217984 (PMC6555508; doi:10.1371/journal.pone.0217984)
Supplement: S4 Table — (DOCX) [file pone.0217984.s004.docx]

**S4 Table. Cox proportional hazard regression analyses for the risk of dementia among patients with benign prostatic hyperplasia**

|  | Univariate model | | |  | Multivariable model | | |
| --- | --- | --- | --- | --- | --- | --- | --- |
|  | HR | (95% CI) | P |  | HR | (95% CI) | P |
| Depression | 1.22 | (0.98‒1.51) | 0.072 |  | 1.22 | (0.98‒1.52) | 0.078 |
| Age (years) |  |  |  |  |  |  |  |
| 50~60 | 0.31 | (0.20‒0.47) | <0.001 |  | 0.33 | (0.21‒0.51) | <0.001 |
| 60~70 | 1.00 |  |  |  | 1.00 |  |  |
| 70~80 | 2.05 | (1.66‒2.54) | <0.001 |  | 1.87 | (1.49‒2.34) | <0.001 |
| >80 | 3.95 | (3.04‒5.14) | <0.001 |  | 3.56 | (2.73‒4.64) | <0.001 |
| Insurance premium (TWD) |  |  |  |  |  |  |  |
| ≥45,801 | 0.36 | (0.22‒0.58) | <0.001 |  | 0.80 | (0.48‒1.33) | 0.392 |
| 28,801–45,800 | 0.56 | (0.38‒0.82) | 0.003 |  | 1.10 | (0.73‒1.64) | 0.647 |
| 15,841–28,800 | 1.00 |  |  |  | 1.00 |  |  |
| <15,840 | 1.64 | (1.36‒1.98) | <0.001 |  | 1.13 | (0.92‒1.38) | 0.234 |
| Dependent | 0.95 | (0.73‒1.23) | 0.675 |  | 0.88 | (0.67‒1.16) | 0.362 |
| Number of outpatient visits | 1.01 | (1.01‒1.02) | <0.001 |  | 1.01 | (1.00‒1.01) | <0.001 |
| Catastrophic illness certificate | 1.67 | (1.32‒2.10) | <0.001 |  | 1.37 | (1.08‒1.74) | 0.009 |
| Hypertension | 1.19 | (0.88‒1.61) | 0.249 |  | 1.20 | (0.89‒1.63) | 0.230 |
| Diabetes | 1.09 | (0.70‒1.69) | 0.697 |  | 1.18 | (0.75‒1.85) | 0.482 |
| Coronary artery disease | 0.53 | (0.14‒1.94) | 0.336 |  | 0.48 | (0.14‒1.66) | 0.248 |
| Hyperlipidemia | 0.68 | (0.22‒2.15) | 0.515 |  | 0.74 | (0.23‒2.38) | 0.617 |
| Cerebrovascular disease | 3.33 | (1.54‒7.19) | 0.002 |  | 2.99 | (1.34‒6.66) | 0.007 |

CI, confidence interval; HR, hazard ratio; TWD, Taiwan dollar
